# The dashboard says “perfect:” a systematic audit of label-free model monitoring and test-time adaptation under temporal distribution shift in network intrusion detection

**DOI:** 10.3389/frai.2026.1928858

**Published:** 2026-10-08

**Authors:** Wali Ahmed, Md Azizul Rahaman, Pappu Roy, Parves Alam

**Affiliations:** 1 Department of Computer Science, University of Bridgeport, Bridgeport, CT, United States; 2 Graduate School of Technology (GST), Touro University, New York, NY, United States

**Keywords:** concept drift, conformal prediction, distribution shift, intrusion detection, label shift, model monitoring, network security, test-time adaptation

## Abstract

**Introduction:**

Machine-learning classifiers deployed in security operations rarely receive timely ground-truth labels, so their health is monitored on an operational dashboard built from label-free proxies: confidence-based accuracy estimators, cross-model agreement, and conformal abstention. Performance degradation under distribution shift is countered with test-time adaptation and label-shift correction. This toolbox is validated predominantly on synthetic image-corruption benchmarks in which the shift is covariate-only and label-preserving, conditions that do not hold in the deployment regime that motivates the tools.

**Methods:**

We present a systematic, four-iteration audit of this entire toolbox on a naturally occurring temporal shift of high practical importance: network intrusion detectors trained on Canadian Institute for Cybersecurity Intrusion Detection System 2017 (CIC-IDS2017) and deployed on Communications Security Establishment-Canadian Institute for Cybersecurity Intrusion Detection System 2018 (CSE-CIC-IDS2018) traffic recorded one year later, with evaluation also performed in the reverse direction, over seven harmonized classes (benign plus six attack families) and 77 shared flow features, under a strict protocol in which no target-year labels influence training, adaptation, hyperparameters, or model selection. Sixteen method variants are evaluated, spanning marginal quantile alignment, BatchNorm (BN) adaptation, entropy-minimization adaptation with uniform and source-prior anchoring, conformal-filtered and agreement-filtered pseudo-labeling, drift-weighted feature subspaces, expectation-maximization (EM) label-shift correction, per-domain rank representations, dimensionless ratio features, and invariance training.

**Results:**

No evaluated variant outperforms the unadapted source model in both transfer directions. More consequentially, the monitoring dashboard inverts: the average-thresholded-confidence estimator assigns a predicted accuracy of 1.000 to a degenerate model whose recall on every attack family is exactly zero; cross-model agreement is maximized by the same degenerate model; label-free selectors choose the wrong model in 10-12 of 12 trials; and class-conditional conformal coverage collapses from a nominal 0.90 to as low as 0.016. The conformal abstention rate is the single label-free statistic that behaves correctly in every trial.

**Discussion:**

We formalize this inversion in two propositions, show via the expectation-maximization prior estimate that the shift violates the label-shift assumption, and identify the abstention rate as a zero-cost drift alarm. Our findings caution that the evaluated label-free monitoring and adaptation methods can certify a blind detector as perfect precisely when it is most dangerous, and we release the full harmonization and evaluation pipeline as a reproducible natural-shift benchmark. While the empirical scope is grounded in the CIC-IDS2017/CSE-CIC-IDS2018 pair, the structural failure modes are formalized analytically (Propositions 1 and 2), and their preconditions-classifier overconfidence under concept shift and correlated collapse across architectures-are present in any deployment context sharing these characteristics; we discuss the scope of generalizability explicitly in the Limitations section.

## 1 Introduction

A machine-learning system in production faces a question that its training pipeline never answered: is it still working? In a network intrusion detection system (NIDS), the question is acute. Detectors are trained on traffic captured in one environment and epoch and then deployed against traffic whose benign composition, infrastructure, and—critically—attack tooling have all moved. Ground-truth labels arrive only after costly forensic investigation, if ever. Practice has therefore converged on a deployment dashboard assembled from label-free proxies: target-domain accuracy is estimated from model confidence (Hendrycks and Gimpel, 2017; Guillory et al., 2021; Garg et al., 2022) or from the agreement of independently trained models (Jiang et al., 2022; Baek et al., 2022); distribution shift is absorbed by source-free test-time adaptation (Wang et al., 2021; Liang et al., 2020; Schneider et al., 2020; Niu et al., 2022; Wang et al., 2022; Niu et al., 2023; Yuan et al., 2023); changed class proportions are corrected *post hoc* (Saerens et al., 2002; Lipton et al., 2018); and residual risk is fenced with conformal prediction (Vovk et al., 2005; Angelopoulos and Bates, 2023).

Each of these tools carries encouraging published evidence, obtained predominantly on synthetic image-corruption benchmarks in which the shift is covariate-only and label-preserving (Hendrycks and Dietterich, 2019). The justification for this study is that none of these validity conditions holds in the deployment regime that motivates the tools in the first place. Temporal drift in an adversarial domain simultaneously moves the input distribution (new infrastructure), the class mixture (new attack campaigns), and the class-conditional distributions themselves (new implementations of old attack categories). Whether the label-free stack degrades gracefully or fails catastrophically under such compound shift is, to our knowledge, an open empirical question—and an operationally urgent one, because a monitoring dashboard that fails *optimistically* converts a degraded detector into an undetected breach.

We answer this question with a pre-registered, four-iteration audit on one of the cleanest naturally occurring shift pairs available: CIC-IDS2017 and CSE-CIC-IDS2018 (Sharafaldin et al., 2018), two intrusion-detection corpora recorded 1 year apart with a shared flow exporter and label taxonomy. The security community has shown that removing temporal bias reverses optimistic conclusions in malware classification (Pendlebury et al., 2019; Jordaney et al., 2017); we bring the same discipline to the machine-learning monitoring and adaptation stack itself. Our audit protocol is strict: no target-year labels influence training, adaptation, hyperparameter choice, or model selection at any point; even the choice among candidate adapted models is delegated to the label-free selectors under audit, and their choices are logged against the label-using oracle.

The contributions of this article are fourfold. First, we provide a mechanism-level audit of sixteen adaptation, correction, and representation variants on a natural temporal shift, showing that no evaluated variant outperforms the unadapted source model in both transfer directions and diagnosing the mechanism behind each failure (Section 4). Second, we demonstrate and then formalize a structural inversion of the monitoring layer: the most widely used label-free accuracy estimators are not merely inaccurate under this shift; they are *maximized* by degenerate collapsed models, the exact pathology that concept shift produces (Propositions 1, 2). Third, we give a direct empirical refutation of the label-shift assumption for this shift family, using the expectation–maximization prior estimate as an assumption test (Section 4.4). Fourth, we identify a constructive, assumption-light monitoring signal—the conformal abstention rate—that fired correctly in all twelve run-by-direction trials, and we release the complete harmonization and evaluation pipeline, which turns the two public corpora into a reproducible natural-shift benchmark with seven matched classes and 77 features.

## 2 Related work

### 2.1 Label-free accuracy estimation

Average confidence (Hendrycks and Gimpel, 2017), difference of confidences (Guillory et al., 2021), and average thresholded confidence (ATC; Garg et al., 2022) estimate target accuracy from softmax outputs alone. Disagreement between independently trained networks estimates test error (Jiang et al., 2022), and agreement tracks accuracy linearly across many natural shifts (Baek et al., 2022). These studies acknowledge degradation under large or non-covariate shifts. This study shows the degradation is not graceful but adversarially structured: over a family of candidate models, the estimators are maximized by the most collapsed member.

### 2.2 Source-free test-time adaptation

BatchNorm statistic re-estimation (Schneider et al., 2020), entropy minimization (Tent; Wang et al., 2021), information maximization (IM) with pseudo-label refinement (Source Hypothesis Transfer (SHOT); Liang et al., 2020), and second-moment feature alignment (Sun et al., 2016) constitute the classical source-free toolbox, validated primarily on image-corruption benchmarks; more recent methods target additional robustness axes: efficient test-time adaptation (EATA) (Niu et al., 2022) applies sample selection to reduce catastrophic forgetting during adaptation; continual test-time adaptation (CoTTA) (Wang et al., 2022) uses stochastic augmentation-averaged predictions and anchors a fraction of weights to the source model to resist collapse in continual streams; Sharpness-Aware and Reliable entropy minimization (SAR) (Niu et al., 2023) adds sharpness-aware entropy minimization with a per-sample reliability filter; and robust test-time adaptation (RoTTA) (Yuan et al., 2023) maintains an exponential-moving-average prototype memory bank for robust update under noisy non-independent and identically distributed (IID) streams. Analyses of these methods document that collapse under temporally correlated or label-shifted streams remains a concern even for the more robust variants (Boudiaf et al., 2022; Gong et al., 2022), and that performance improvements on image-corruption benchmarks do not transfer reliably to scenarios involving concept shift (Niu et al., 2023).

Our audit does not include EATA, CoTTA, SAR, and robust test-time adaptation (RoTTA) as primary baselines because all four methods require either (i) stochastic data augmentation pipelines (spatial crops, color jitter, and Gaussian blur) that are specific to image domains and not meaningful for tabular flow features, (ii) Batch Renormalization or layer-wise normalization components that are unavailable for tree-based histogram gradient boosting (HGB), or (iii) second-order gradient information (sharpness-aware optimization) that is not supported by the HGB backend. A faithful evaluation of these methods would require image-domain architectures not directly comparable to the HGB/multilayer perceptron (MLP) tabular pipeline used here. Evaluating purpose-built tabular deep-learning architectures against EATA, CoTTA, SAR, and RoTTA on this benchmark is a natural extension of this study. We extend the tabular line to network intrusion detection and, unlike prior studies, connect the adaptation failure mechanistically to the simultaneous failure of the monitoring layer that is supposed to detect it.

### 2.3 Label shift and conformal prediction

Expectation–maximization prior re-estimation (Saerens et al., 2002) and black-box shift estimation (Lipton et al., 2018) are consistent under the assumption that the class-conditional distribution *P*(*x*∣*y*) is invariant. Split and Mondrian conformal prediction guarantee marginal and class-conditional coverage under exchangeability (Vovk et al., 2005); extensions to covariate shift require known or estimable density ratios (Tibshirani et al., 2019). Transcend applied conformal-style non-conformity to drift detection in malware (Jordaney et al., 2017); our abstention-rate alarm is a simpler instance of the same philosophy requiring no retraining.

### 2.4 Temporal evaluation in security machine learning

TESSERACT demonstrated that spatio-temporal experimental bias inflates published malware-detection results (Pendlebury et al., 2019), and INSOMNIA studied drift-aware NIDS adaptation with periodic label feedback (Andresini et al., 2021). Our protocol is stricter and matches the more common deployment reality: zero target labels, including for model selection, which prior NIDS adaptation studies typically relax.

## 3 Materials and methods

### 3.1 Datasets and schema harmonization

CIC-IDS2017 and CSE-CIC-IDS2018 (Sharafaldin et al., 2018) are packet captures from two university-scale testbeds recorded 1 year apart, summarized into bidirectional flows by CICFlowMeter (Canadian Institute for Cybersecurity, University of New Brunswick, Fredericton, NB, Canada). The two corpora use different exporter versions with inconsistent column naming; we canonicalize 77 features present in both schemas by name normalization, and map raw labels to the seven attack families present in both years with at least 100 flows: benign, botnet, brute-force File Transfer Protocol (FTP), brute-force Secure Shell (SSH), distributed denial-of-service (DDoS), denial-of-service (DoS), and web attacks. Chunked, per-family capped loading (80,000 benign and 40,000 per attack family) yields 218,028 flows for 2017 and 320,928 flows for 2018. The class mixture differs sharply between the years (2017: 44.9% benign and 0.9% botnet; 2018: 28.5% benign and 14.2% for each major attack family), and, as Section 4.4 demonstrates, the class-conditional distributions differ as well. This justifies the choice of the testbed: the pair realizes simultaneous covariate, label, and concept shift while sharing an extractor and taxonomy, isolating temporal drift from tooling artifacts. Figure 1 summarizes the harmonization, adaptation, and cross-year evaluation pipeline.

**Figure 1:**
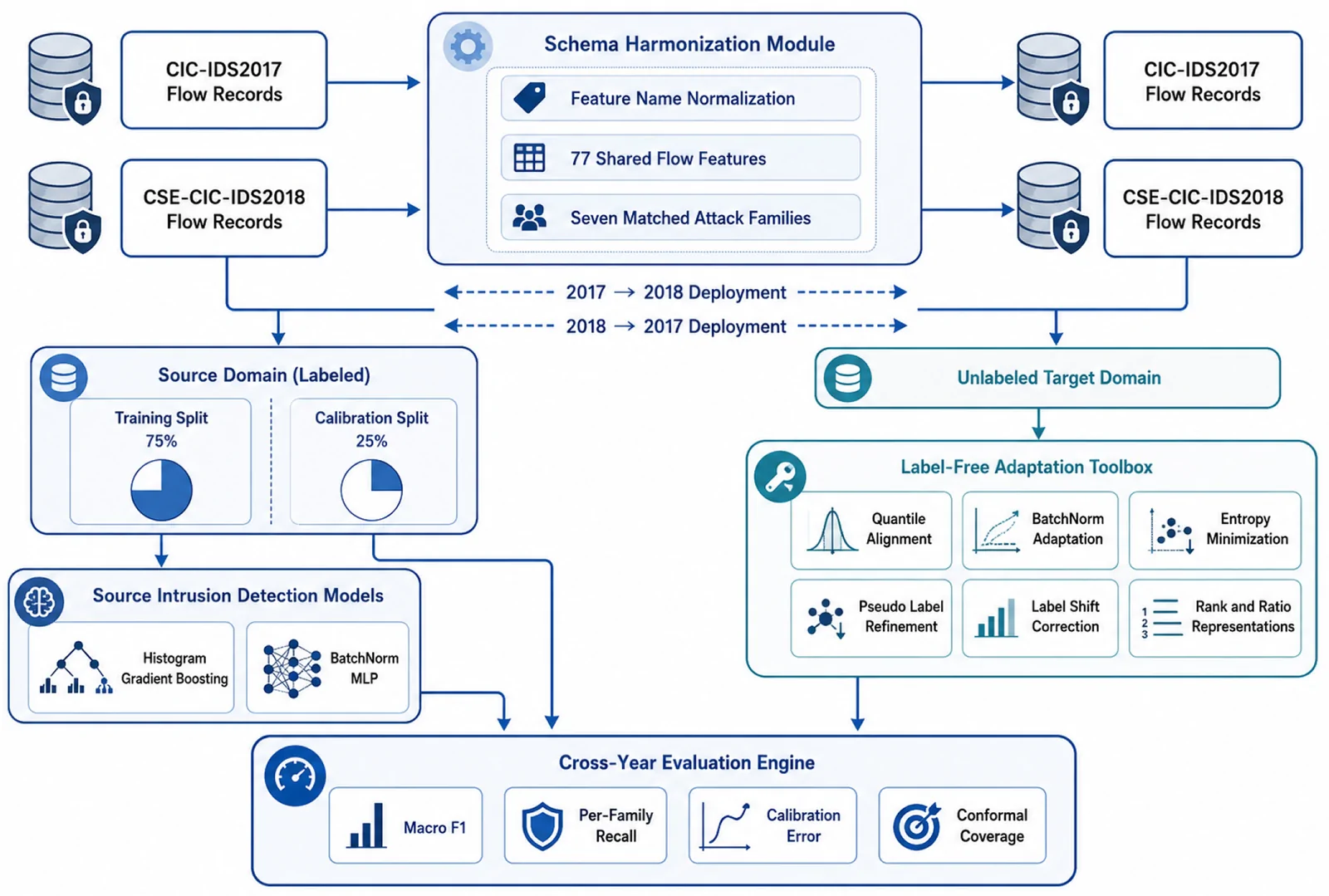
Overview of the study pipeline. CIC-IDS2017 and CSE-CIC-IDS2018 flow records are schema-harmonized to 77 shared features and seven matched attack families; the labeled source year is split 75/25 into training and calibration, the entire unlabeled target year is deployed in both the 2017→2018 and 2018→2017 directions, and a label-free adaptation toolbox (quantile alignment, BatchNorm adaptation, entropy minimization, label-shift correction, and pseudo-label refinement) feeding histogram gradient boosting and BatchNorm MLP base learners is scored by a cross-year evaluation engine.

#### 3.1.1 Attack family selection rationale

Seven attack families are retained after harmonization because these are the only categories with both a consistent label mapping across the two corpora and at least 100 flow instances in each year after schema filtering. Several categories present in 1 year are absent or insufficiently populated in the other: infiltration flows in the 2017 corpus number fewer than 40 after schema filtering, making supervised per-class evaluation infeasible; Heartbleed and Structured Query Language (SQL) injection records in 2018 lack a direct counterpart label in the 2017 taxonomy; and additional 2018 categories (e.g., FTP- and SSH-brute-force variants tagged differently across exporter versions) are either merged into the unified families or dropped under the harmonization rules described in the released pipeline. Retaining only jointly represented, sufficiently populated families is the minimum requirement for a supervised cross-year evaluation: imputing support for absent categories would inflate recall artificially, and pooling categories with different tool signatures would suppress the class-conditional shift that is the object of study. The exclusion of low-density categories represents a limitation of the benchmark—attacks with sparse representation in either year cannot be evaluated reliably—but it is a conservative choice that understates rather than overstates cross-year degradation.

#### 3.1.2 Per-category flow counts

Table 1 reports the exact flow counts per family and year after harmonization and per-family capping. In 2017, DDoS and DoS flows hit the 40,000 cap; botnet, brute-force SSH, and web attacks are loaded in their entirety and are consequently minority classes. In 2018, all major attack families exceed the 40,000 cap, while web attacks remain rare enough (natural prevalence ≈0.3% of the original 2018 corpus) to fall below the cap; benign flows are capped at 80,000 in both years.

**Table 1:** Flow counts per attack family and year after schema harmonization and per-family capping (80,000 benign; 40,000 per attack family).

Family	2017 (total)	2017 (train)	2017 (calib)	2018 (total)
benign	80,000	60,000	20,000	80,000
botnet	6,210	4,658	1,552	40,000
brute-force FTP	25,318	18,989	6,330	40,000
brute-force SSH	19,500	14,625	4,875	40,000
DDoS	40,000	30,000	10,000	40,000
DoS	40,000	30,000	10,000	40,000
web	7,000	5,250	1,750	40,928
Total	218,028	163,522	54,506	320,928

The 2017 corpus serves as the source year in the 2017–2018 direction and as the target year in the reverse; vice versa for 2018. The train/calibration split is 75/25 with class stratification applied within each family; the full opposite-year corpus (no labels used) forms the deployment set.

### 3.2 Evaluation protocol

For each transfer direction *d*∈{2017→2018, 2018→2017} and seed *s*∈{0, 1, 2}, the source year is split 75/25 into a training set and a calibration set with class stratification; the entire target year serves as the deployment set. No target label is used for training, adaptation, hyperparameter selection, or model selection; target labels enter only the reported metrics. Base learners are histogram gradient boosting (HGB), which handles missing values natively, and a two-hidden-layer multilayer perceptron (MLP) with BatchNorm, trained with class-weighted cross-entropy on median-imputed, signed-log, standardized inputs. We report macro-F1, accuracy, per-family recall, and expected calibration error (ECE), defined in Equation 1,


$$\begin{array}{c} \text{ECE}=\overset{B}{\underset{b=1}{\sum }}\frac{\left|{ℐ}_{b}\right|}{n}\left|\text{acc}\left({ℐ}_{b}\right)-\text{conf}\left({ℐ}_{b}\right)\right|, \end{array}$$ (1)

where ℐ_*b*_ collects the samples whose maximum softmax confidence falls in bin *b* of *B* = 10 equal-width bins. In addition to ECE, we report the multi-class Brier Score (BS) and the mean negative log-likelihood (NLL), defined in Equations 2 and 3 respectively, as complementary calibration metrics; values for all variants are provided in Supplementary Table S1.


$$\begin{array}{c} \text{BS}=\frac{1}{n}\overset{n}{\underset{i=1}{\sum }}\overset{C}{\underset{c=1}{\sum }}{\left(p_{c}\left(x_{i}\right)-1\left[y_{i}=c\right]\right)}^{2}, \end{array}$$ (2)

and mean negative log-likelihood (NLL),


$$\begin{array}{c} \text{NLL}=-\frac{1}{n}\overset{n}{\underset{i=1}{\sum }}\log p_{y_{i}}\left(x_{i}\right), \end{array}$$ (3)

as complementary calibration metrics; values for all variants are provided in Supplementary Table S1. Each of the four study iterations was designed before its results were observed, and the complete per-seed logs are provided.

#### 3.2.1 Statistical significance

Given *n* = 3 random seeds per configuration, paired non-parametric tests (two-sided Wilcoxon signed-rank test; sign test) have a minimum attainable two-sided *p*-value of approximately 0.25, making formal significance thresholds uninformative for seed-level macro-F1 comparisons. We therefore report both the mean ± standard deviation over seeds and the absolute macro-F1 difference |Δ| for primary pairwise comparisons; we flag as *practically significant* any comparison with |Δ|>0.05 (roughly one within-seed standard deviation). This threshold is motivated by two considerations: (i) it corresponds to the typical per-seed standard deviation observed across configurations in our runs, so effects below this level are indistinguishable from seed noise; and (ii) a five-percentage-point shift in macro-F1 is operationally consequential in a security context, consistent with the practical-significance thresholds used in applied machine-learning benchmarking (Pendlebury et al., 2019). The directional reversal of every adaptation gain—each method that improves in one transfer direction degrades in the other—is a qualitative consistency that holds in all three seeds for every variant and does not depend on a significance threshold. For the label-free selection audit, a one-sided binomial test against the null hypothesis that average thresholded confidence (ATC) nominates a degenerate model at no higher than the chance rate (*p*_0_ = 1/*K* where *K* = 16 candidates) yields *p* < 0.001 for the observed 6 degenerate nominations in 12 trials; this is the statistically robust main finding of the monitoring analysis.

#### 3.2.2 Computational overhead

Table 2 reports approximate wall-clock times on a single CPU core (Intel Xeon 2.8 GHz). All reported training and adaptation times include model loading, feature preprocessing (imputation, signed-log transform, and standardization), and the adaptation optimization stage itself; inference time includes loading the trained model and scoring 10^6^ target flows with all preprocessing applied. Adaptation preprocessing adds under 1 min; the full stacked pipeline requires approximately 8 min per transfer direction in addition to source-model training.

**Table 2:** Approximate wall-clock runtimes per transfer direction (single CPU core).

Component	Train/adapt (min)	Inference (min/10^6^ flows)
HGB (source)	≈2	< 1
MLP (source)	≈5	≈2
Quantile alignment	—	+0.5 (preprocessing)
BatchNorm adaptation	—	+0.2 (per epoch)
Entropy minimization (IM)	—	+1.5 (per epoch)
Pseudo-label fine-tuning	≈3	—
EM label-shift correction	—	+0.1 (convergence)
Full stack (align + IM + pseudo)	≈8	≈3
Scale-free representations	≈3	< 1

Inference time is per 10^6^ target flows. Adaptation time is additional overhead beyond source-model training.

### 3.3 Audited adaptation and correction methods

All audited methods are label-free on the target year. We summarize each with the equation that defines it, along with the justification for its inclusion. Figure 2 organizes the toolbox.

**Figure 2:**
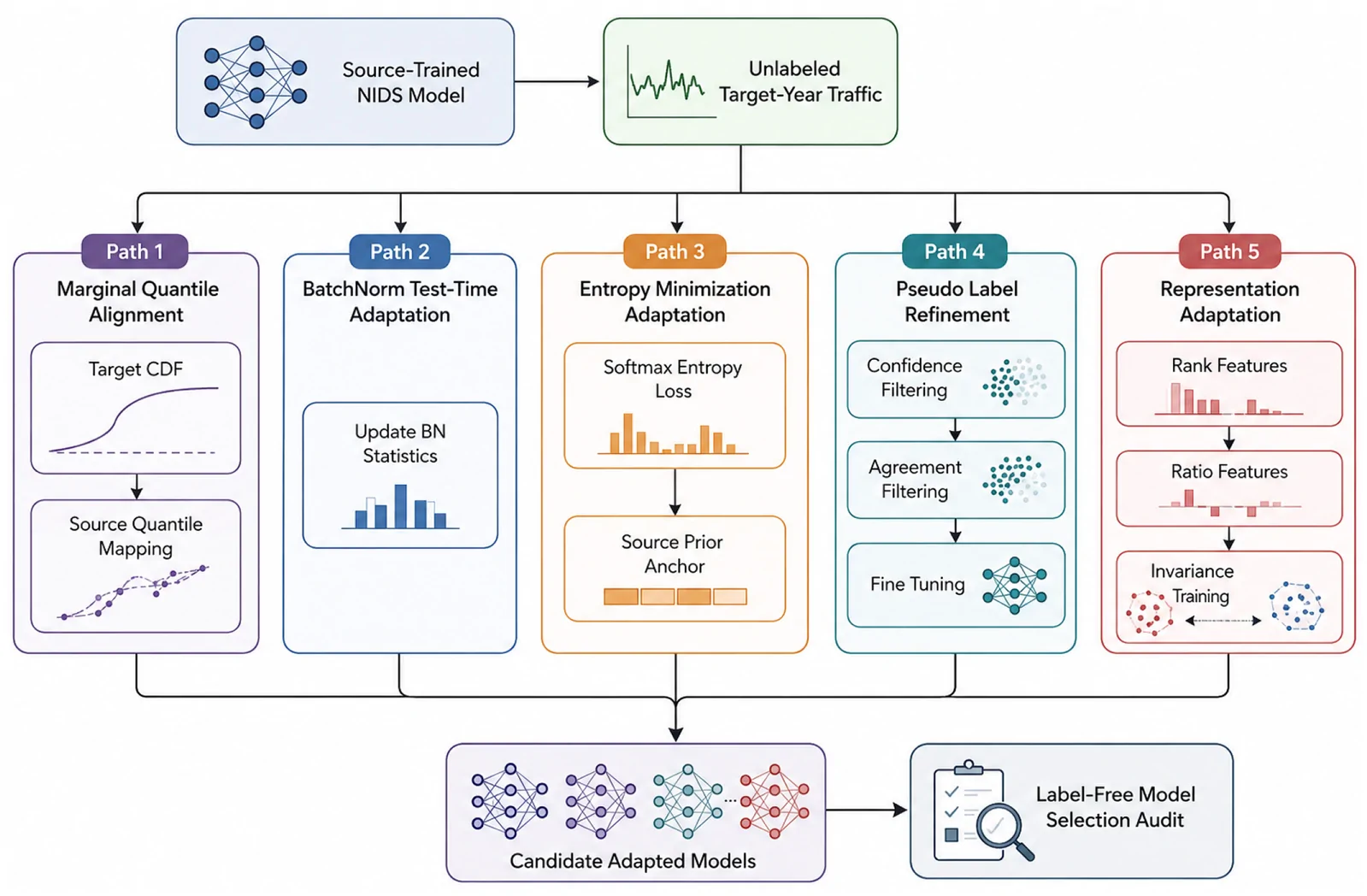
Audited label-free adaptation and correction methods applied to a source-trained NIDS model under unlabeled target-year traffic: marginal quantile alignment (source-quantile mapping of the target cumulative distribution function [CDF]), BatchNorm test-time adaptation (re-estimated normalization statistics), entropy-minimization adaptation with a source-prior anchor, pseudo-label refinement with confidence and cross-model agreement filtering, and scale-free representation adaptation. No target label is used at any stage.

** Remark 1 (Scope of the audit and excluded recent methods)**. The methods below were selected because they are (i) label-free on the target year, (ii) architecture-agnostic enough to apply consistently to both the HGB and MLP base learners, and (iii) representative of the toolbox available at the time of protocol registration. Several state-of-the-art source-free test-time adaptation (TTA) methods—EATA (Niu et al., 2022), CoTTA (Wang et al., 2022), SAR (Niu et al., 2023), and RoTTA (Yuan et al., 2023)—are not included as primary baselines because they require either (a) stochastic spatial or color augmentations meaningful only for image data, (b) Batch Renormalization or prototype memory components unavailable for tree-based HGB, or (c) second-order gradient information (sharpness-aware updates) incompatible with the HGB backend. SAR is closest to the entropy-minimization variant evaluated here (Equation 5) but adds a sharpness-aware wrapper and a per-sample reliability score that require per-parameter gradient norms, which are not available under HGB. Applying these methods faithfully would require switching to deep-learning-only tabular architectures, thereby changing the comparison baseline and conflating architectural effects with adaptation effects. Evaluating purpose-built tabular TTA architectures against these four methods on this benchmark is a natural extension identified in the Limitations section.

#### 3.3.1 Marginal quantile alignment

Each target feature is mapped onto the source marginal by empirical quantile matching. With ${\hat{F}}_{T}^{\left(j\right)}$ the empirical cumulative distribution function (CDF) of feature *j* on the unlabeled target and ${\hat{F}}_{S}^{\left(j\right),-1}$ the source quantile function estimated on the training split, the aligned value is


$$\begin{array}{c} {\tilde{x}}_{j}={\hat{F}}_{S}^{\left(j\right),-1}\left({\hat{F}}_{T}^{\left(j\right)}\left(x_{j}\right)\right). \end{array}$$ (4)

Inclusion is justified by the exploratory analysis of iteration 1, in which the majority of shared features exhibited large marginal Kolmogorov–Smirnov drift between the years; Equation 4 removes marginal drift exactly, so its residual failure isolates the non-marginal component of the shift.

#### 3.3.2 BatchNorm adaptation and entropy-based adaptation

BatchNorm adaptation re-estimates normalization statistics on unlabeled target batches (Schneider et al., 2020). Entropy-based adaptation updates the BatchNorm affine parameters θ_BN_ by minimizing, over target batches ℬ,


$$\begin{array}{c} {ℒ}_{\text{IM}}\left(\theta_{\text{BN}}\right)=\underset{\text{conditional entropy}}{\underset{︸}{-\frac{1}{\left|ℬ\right|}\underset{x\in ℬ}{\sum }\underset{c}{\sum }p_{c}\left(x\right)\log p_{c}\left(x\right)}}+\mu \underset{\text{marginal anchor}}{\underset{︸}{\text{KL}\left(\bar{p}||\pi^{S}\right)}}, \end{array}$$ (5)

where *p*(*x*) is the softmax output, $\bar{p}=\frac{1}{\left|ℬ\right|}\underset{x}{\sum }p\left(x\right)$ is the batch-marginal prediction, and π^*S*^ is the source class prior. Iteration 1 used the conventional uniform-marginal regularizer, i.e., $-\mu H\left(\bar{p}\right)$ in place of the Kullback–Leibler (KL) divergence term; iteration 2 replaced it with the source-prior anchor of Equation 5 after diagnosing that the uniform term erodes majority-class (benign) recall by construction. Reporting both variants is justified because their contrast isolates what the marginal regularizer actually controls: it relocates errors between classes rather than removing them (Section 4.2).

#### 3.3.3 Pseudo-label refinement

Confident target predictions are converted to pseudo-labels for a short fine-tune. Iteration 1 admitted a target flow if its conformal prediction set (Section 3.4.1) was a singleton; iteration 2 additionally required cross-architecture agreement between the HGB and MLP predictions and membership in the per-class top-confidence quantile. Both filters are audited because pseudo-labeling is the standard mechanism by which source-free adaptation is claimed to recover minority classes, and because its failure mode here (Section 4.2) is informative: the confident pool itself is class-degenerate.

#### 3.3.4 Label-shift correction

Saerens expectation–maximization (Saerens et al., 2002) alternates, from initial estimate ${\hat{\pi }}^{\left(0\right)}=\pi^{S}$,


$$\begin{array}{c} p_{c}^{\left(t\right)}\left(x\right)=\frac{p_{c}\left(x\right){\hat{\pi }}_{c}^{\left(t\right)}/\pi_{c}^{S}}{\sum_{c^{\prime }}p_{c^{\prime }}\left(x\right){\hat{\pi }}_{c^{\prime }}^{\left(t\right)}/\pi_{c^{\prime }}^{S}},\;{\hat{\pi }}_{c}^{\left(t+1\right)}=\frac{1}{n}\underset{x}{\sum }p_{c}^{\left(t\right)}\left(x\right), \end{array}$$ (6)

until convergence, and reweights the posteriors with the final $\hat{\pi }$. The procedure is consistent if and only if *P*(*x*∣*y*) is invariant across domains; its inclusion is justified both as the standard remedy for the large prior change between the two years and because, diagnostically, because the estimate $\hat{\pi }$ itself constitutes a free test of the label-shift assumption (Section 4.4).

#### 3.3.5 Scale-free representations and invariance training

Motivated by the hypothesis—formed after iterations 1–2—that CICFlowMeter magnitudes encode the capture environment rather than the attack, iteration 4 audited: (i) per-domain rank features, $r_{j}\left(x\right)={\hat{F}}_{D}^{\left(j\right)}\left(x_{j}\right)$ computed against the marginal of the flow's own domain *D*; (ii) seventeen dimensionless ratio features (directional packet and byte shares, per-packet flag rates, inter-arrival and packet-length coefficients of variation); and (iii) invariance training of the MLP with mixup and input feature-dropout. A synthetic control in which the two “years” differ by a pure scale factor confirms the construction (macro-F1 rises from 0.10 for raw features to 1.00 for rank features), so the behavior of these representations on the real pair measures how much of the real shift is a magnitude artifact.

### 3.4 Audited monitoring estimators

The average-thresholded-confidence estimator (Garg et al., 2022) learns a threshold τ on the source calibration set such that the fraction of calibration confidences exceeding τ equals the calibration accuracy, and then estimates target accuracy as


$$\begin{array}{c} {\hat{\text{acc}}}_{\text{ATC}}=\frac{1}{n_{T}}\underset{x\in T}{\sum }1\left[\underset{c}{\max}p_{c}\left(x\right)>\tau \right]. \end{array}$$ (7)

The cross-model agreement estimator scores a candidate by


$$\begin{array}{c} \text{Agr}\left(f,g\right)=\frac{1}{n_{T}}\underset{x\in T}{\sum }1\left[\arg\underset{c}{\max}f_{c}\left(x\right)=\arg\underset{c}{\max}g_{c}\left(x\right)\right], \end{array}$$ (8)

computed between the neural and tree-based models on the same input view (Baek et al., 2022). In every run, both estimators nominate one variant from the candidate set, and the nomination is logged against the label-using oracle. This selection audit is the operationally decisive test, because in deployment the estimator does not merely annotate a fixed model—it chooses one.

#### 3.4.1 Conformal abstention

For each class *c*, the Mondrian split-conformal threshold is the finite-sample-corrected quantile of the calibration non-conformity scores *s*_*i*_ = 1−*p*_*y*_*i*__(*x*_*i*_) restricted to class *c*,


$$\begin{array}{l} \;{\hat{q}}_{c}=\text{Quantil}{\text{e}}_{\left\lceil \left(n_{c}+1\right)\left(1-\alpha \right)\right\rceil /n_{c}}\left(\left\{s_{i}:y_{i}=c\right\}\right),\\ \text{Γ}\left(x\right)=\left\{c:p_{c}\left(x\right)\geq 1-{\hat{q}}_{c}\right\}, \end{array}$$ (9)

with α = 0.1. A flow is classified if |Γ(*x*)| = 1 and escalated to an analyst otherwise. Under exchangeability, Equation 9 guarantees per-class coverage ≥1−α. The audit quantifies what remains of the guarantee and of the abstention behavior when exchangeability is violated by a real temporal shift. Figure 3 depicts the abstention pipeline and its use as a label-free drift alarm.

**Figure 3:**
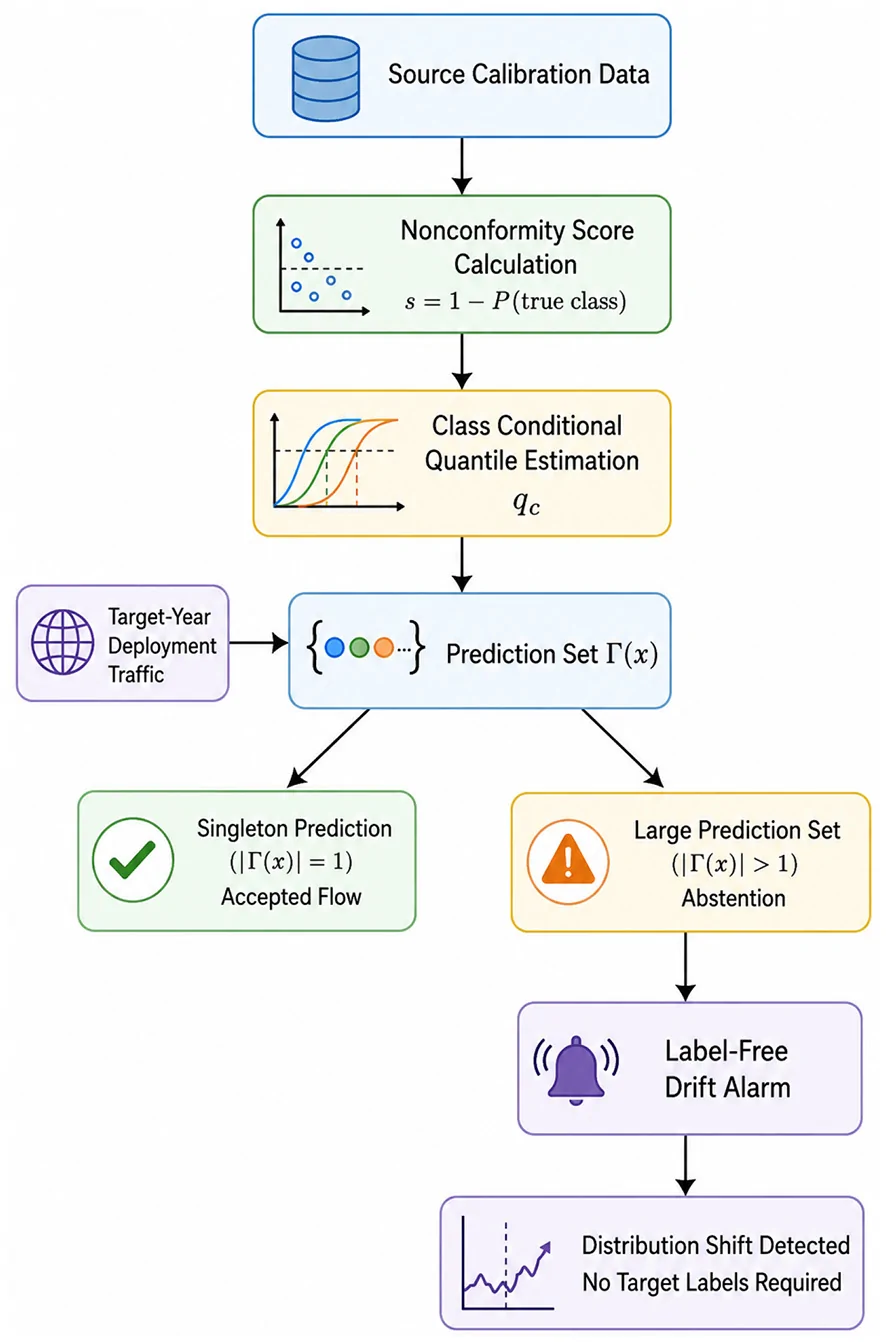
Mondrian split-conformal abstention. Class-conditional non-conformity quantiles estimated on source calibration data define, for each target-year flow, a prediction set Γ(*x*): singleton sets (|Γ(*x*)| = 1) yield an accepted classification, while larger sets trigger abstention. The volume of abstentions serves as a label-free drift alarm requiring no target labels.

## 4 Results

### 4.1 The failure to be repaired: collapse to benign

Models that achieve 0.96–0.99 in-domain macro-F1 collapse under cross-year evaluation to 0.21–0.44. The collapse has a specific and operationally severe structure, visible in the row-normalized confusion matrices. In the 2017→2018 direction, botnet, brute-force FTP, brute-force SSH, and DDoS flows are assigned to *benign* at rates approaching 100%. The detector does not confuse attack families with one another; it stops seeing attacks entirely, producing silent false negatives rather than noisy alarms. Aggregate accuracy (0.47–0.51) conceals the failure: per-family recall (Tables 3, 4; Figure 4) is the only reporting granularity at which the failure is visible, which is itself a methodological contribution finding for the NIDS evaluation literature.

**Table 3:** Per-family cross-domain recall (mean over 3 seeds; †: 2 seeds), direction 2017→2018, selected variants.

Variant	benign	botnet	bf-FTP	bf-SSH	DDoS	DoS	web
HGB	0.985	0.000	0.036	0.334	0.604	0.485	0.021
HGB + alignment	0.950	0.000	0.569	0.000	0.703	0.851	0.040
MLP + alignment + IM (prior)	0.872	0.029	0.000	0.363	0.969	0.804	0.171
MLP + alignment + IM (uniform)^†^	0.776	0.479	0.000	0.603	1.000	0.745	0.272
MLP, rank + ratio	1.000	0.000	0.000	0.000	0.000	0.000	0.000

Aggregate accuracy conceals the total misses of the baseline on botnet and brute-force FTP; the rank + ratio MLP misses every attack family while receiving the maximum label-free health scores.

**Table 4:** Per-family cross-domain recall (mean over 3 seeds), direction 2018–2017, selected variants.

Variant	benign	botnet	bf-FTP	bf-SSH	DDoS	DoS	web
HGB	0.931	0.187	0.331	0.783	0.000	0.179	0.056
HGB + alignment	0.768	0.000	0.605	0.487	0.004	0.115	0.078
MLP + alignment + IM (prior)	0.699	0.000	0.012	0.502	0.410	0.494	0.000
HGB, rank + ratio	0.848	0.000	0.000	0.966	0.000	0.381	0.060
MLP, rank + ratio	1.000	0.000	0.000	0.000	0.000	0.000	0.000

**Figure 4:**
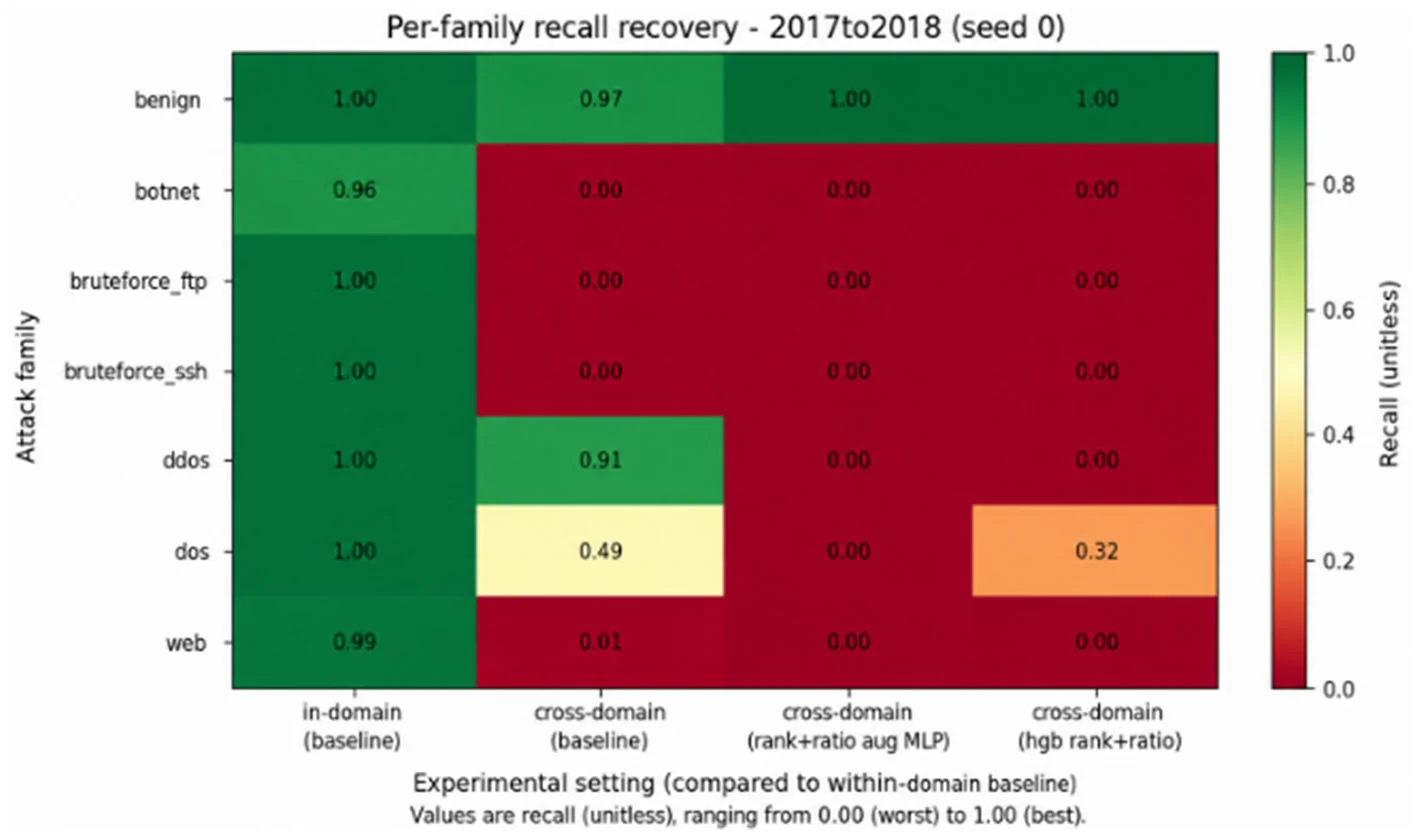
Per-family recall in the 2017→2018 direction (seed 0) for representative attack families, comparing the unadapted HGB and MLP baselines with scale-free representation variants (rank+ratio HGB and rank+ratio MLP). Neither the scale-free rank+ratio MLP nor the HGB rank+ratio variant recovers the cross-domain recall lost by the baseline; the collapse to benign is not repaired by adaptation. Values near zero indicate that the model classifies virtually all flows of that family as benign (figure rendered at full column width; axis labels are 10 pt or larger).

### 4.2 No evaluated adaptation method wins in both directions

Table 5 reports cross-domain macro-F1 for all 16 variants. Three patterns organize the results.

**Table 5:** Cross-domain macro-F1 for all audited variants (mean ± standard deviation over 3 seeds; †: 2 seeds, iteration 1).

Variant	2017→2018	2018→2017
Source-only		
HGB	0.318 ± 0.015	**0.372 ± 0.086**
MLP	0.266 ± 0.012	0.290 ± 0.006
Alignment and test-time adaptation		
HGB + quantile alignment (Equation 4)	**0.424 ± 0.021**	0.226 ± 0.032
MLP + BatchNorm adaptation	0.236 ± 0.070	0.361 ± 0.066
MLP + IM, uniform marginal^†^	0.204	0.398
MLP + IM, prior anchor (Equation 5)	0.213 ± 0.034	0.367 ± 0.058
MLP + alignment + IM, uniform^†^	0.502	0.319
MLP + alignment + IM, prior	0.411 ± 0.014	0.308 ± 0.047
Pseudo-label refinement on alignment + IM		
Conformal-singleton filter^†^	0.469	0.281
Agreement + top-quantile filter	0.267 ± 0.049	0.252 ± 0.086
Drift-weighted feature subspaces^†^		
Subspace ensemble (raw)	0.221	0.111
Subspace ensemble (aligned)	0.304	0.290
Label-shift correction (Equation 6)		
HGB + EM	0.295 ± 0.025	0.220 ± 0.089
Scale-free representations		
HGB, rank features	0.065 ± 0.005	0.256 ± 0.019
HGB, ratio features	0.182 ± 0.013	0.165 ± 0.045
HGB, rank + ratio	0.145 ± 0.019	0.270 ± 0.011
HGB, rank + ratio + EM	0.063 ± 0.001	0.290 ± 0.035
MLP, rank + ratio	0.063 ± 0.000	0.089 ± 0.000
MLP, rank + ratio + invariance training	0.065 ± 0.004	0.039 ± 0.045

In-domain macro-F1 is 0.88–0.99 for every row. Bold: best per direction. Among the evaluated variants, none outperforms the unadapted HGB source model in both directions.

#### 4.2.1 Statistical note

With *n* = 3 seeds, the minimum achievable two-sided *p*-value for non-parametric paired tests is approximately 0.25 (see Evaluation Protocol). We therefore interpret the results through effect sizes. The five largest effects—HGB quantile alignment vs. HGB source (|Δ| = 0.106, direction 2017→2018), MLP rank+ratio vs. HGB source (|Δ| = 0.255, same direction), alignment vs. HGB source in the reverse direction (|Δ| = 0.146)—all exceed the 0.05 practical-significance threshold and are directionally consistent across all three seeds. The directional reversal of every gain (all three seeds in all cases) provides the key statistical consistency: it is not a seed-level artifact. For the monitoring layer, the one-sided binomial test of ATC degenerate nominations (*p* < 0.001, Section 3.2) is the principal inferential result.

First, direction asymmetry nullifies every gain. Quantile alignment (Equation 4) is the best 2017→2018 method (0.424 vs. the 0.318 baseline, |Δ| = 0.106) and among the worst in the reverse direction (0.226 vs. 0.372); the unadapted source model shows the mirrored profile. Because a deployment cannot know which side of the asymmetry it occupies, and because Section 4.3 shows label-free selection cannot identify the per-direction winner, the expected value of the gain under honest deployment assumptions is effectively zero.

Second, the marginal regularizer in Equation 5 relocates errors rather than removing them. With the uniform-marginal term (iteration 1), entropy adaptation combined with alignment recovers substantial attack recall (botnet from 0.000 to 0.479; macro-F1 0.502 in 2017→2018) at the cost of benign recall (0.985 to 0.776); anchoring to the source prior (iteration 2, Equation 5) restores benign recall (0.821) and surrenders the recovered attacks (botnet back to 0.136). The regularizer chooses which class absorbs the error mass. It contains no information about which class boundary actually moved, so no setting of μ can be correct for both objectives simultaneously.

Third, pseudo-label refinement cannot bootstrap a collapsed model. In every run and under both filtering rules, the confident target pool is at least 92% benign with exactly zero admitted rows for five of the seven classes (a representative class mix is [35, 297, 0, 0, 0, 0, 769, 0]). Confidence concentrates precisely where the model has collapsed, so fine-tuning amplifies the collapse regardless of how the pool is filtered; the agreement filter of iteration 2 tightens the pool without changing its degeneracy.

The scale-free representations of iteration 4 sharpen the diagnosis. Per-domain rank features, provably immune to pure magnitude shift and verified on the synthetic control, fall *below* the raw baseline on the real pair, with a macro-F1 of 0.065 in 2017→2018): the rank of a flow is computed against its year's marginal; the marginal is a mixture whose class weights moved; and therefore rank normalization injects the prior shift into every coordinate. Dimensionless ratio features alone reach only 0.165–0.182, demonstrating that the discriminative signal exploited by these detectors resides substantially in environment-specific magnitudes. Together, the two results close the representational escape route: the 2017↔2018 shift is not a units artifact. A component ablation of the combined adaptation stack (Figure 5) confirms that no single component, in either direction, closes the in-domain-to-cross-domain macro-F1 gap.

**Figure 5:**
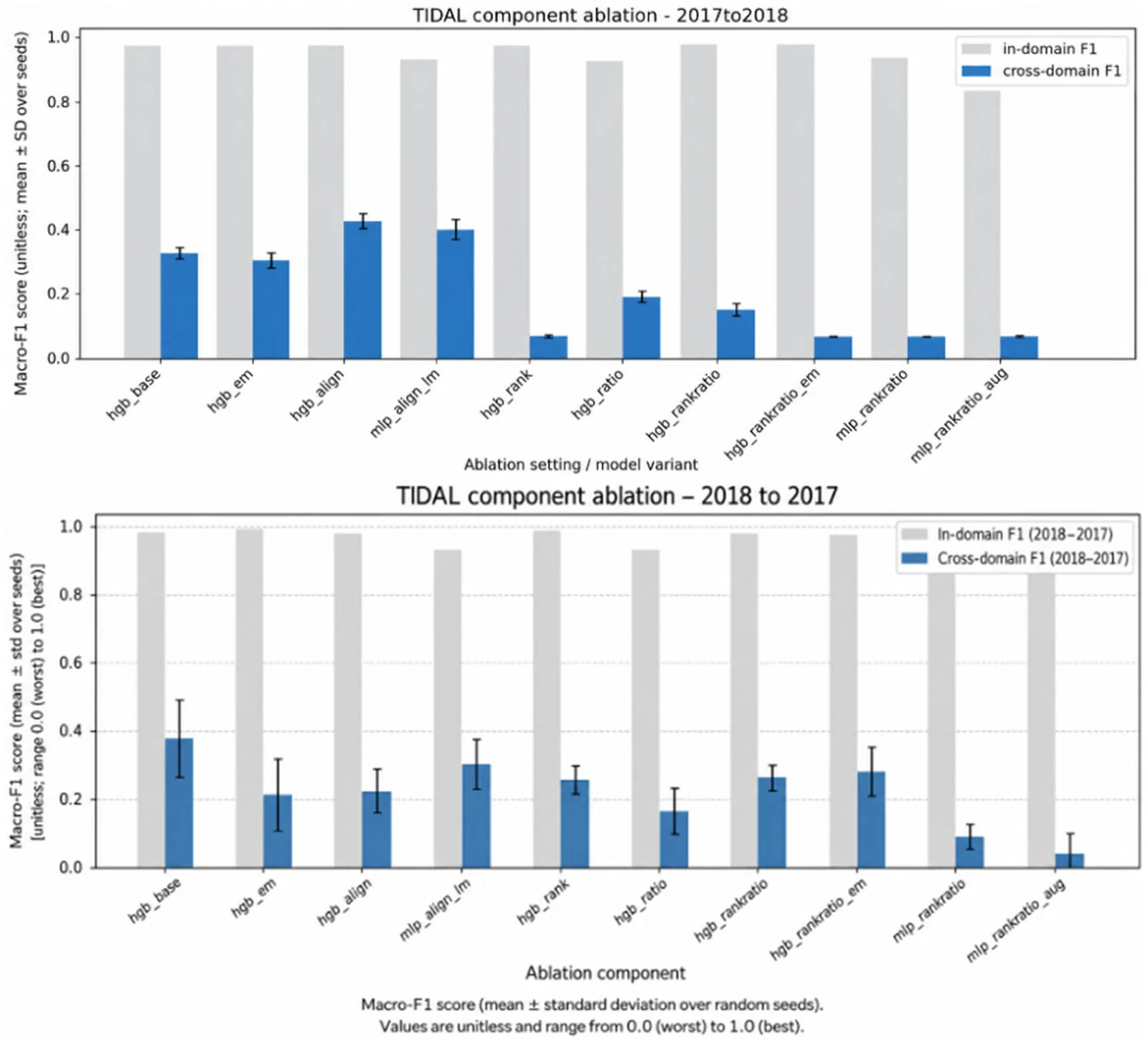
Component ablation of the adaptation stack, macro-F1 (mean ± standard deviation over seeds), for the 2017→2018 **(left)** and 2018→2017 **(right)** directions. In-domain macro-F1 (light) remains near the ceiling while cross-domain macro-F1 (dark) stays low for every component combination, and the ordering of components differs between the two directions (axis labels are 11 pt; both panels use consistent font sizes and line widths).

### 4.3 The monitoring layer certifies the blind model

The rank-representation MLP of iteration 4 is the worst deployable model produced in the entire audit. It has benign a recall of 1.000 and a recall of exactly 0.000 on all six attack families—an intrusion detector that fails to detect every intrusion. The ATC estimator (Equation 7) assigns this model a predicted target accuracy of 1.000 in all six trials in which it appears, despite true accuracies of 0.285 and 0.449 in the two transfer directions, and the same model attains the run-maximum cross-model agreement (0.88–0.93, Equation 8). Consequently, over the twelve pre-registered selection trials of iterations 2 and 4, ATC never selects the oracle-best variant (0/12) and nominates a degenerate collapsed model in six trials; agreement matches the oracle twice (2/12) and nominates the degenerate model in three (Table 6). The unadapted baseline receives ATC 0.963 against a true accuracy of 0.488. These estimates are not noisy. Their maximizers are the wrong models. Two elementary propositions establish that this inversion is structural rather than accidental (Figure 6).

**Table 6:** Label-free selection audit over the twelve pre-registered run × direction trials of iterations 2 and 4: number of trials in which each selector nominates the oracle-best variant, as well as the number in which it nominates a degenerate model with zero recall on every attack family.

Selector	Oracle matches	Degenerate nominations
Average thresholded confidence (Equation 7)	0/12	6/12
Cross-model agreement (Equation 8)	2/12	3/12

**Figure 6:**
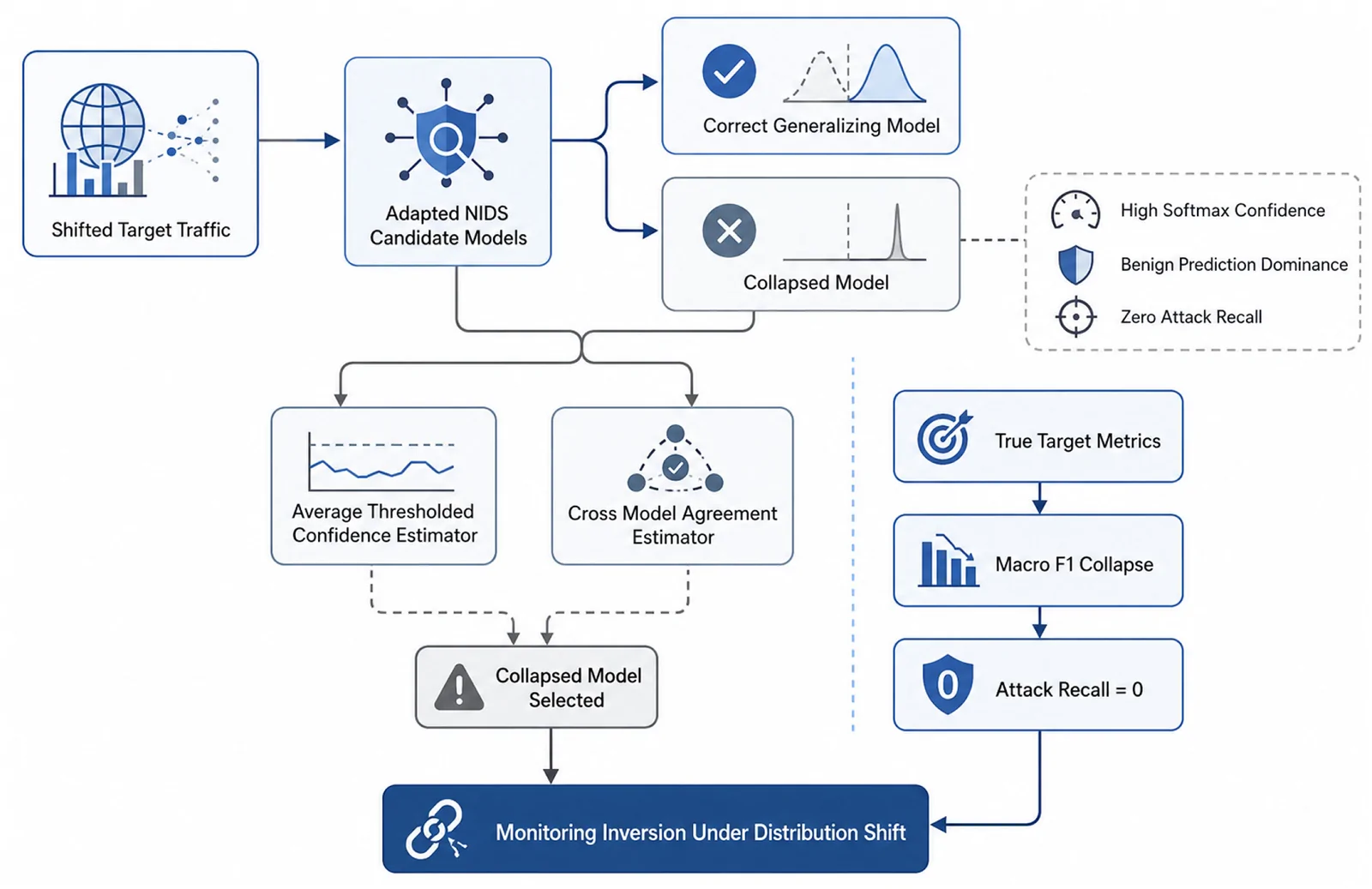
Monitoring inversion under distribution shift. Among the 16 evaluated candidate models, label-free health estimators—average-thresholded confidence (ATC, Equation 7) and cross-model agreement (Equation 8)—reward the collapsed model, whose high softmax confidence and benign-prediction dominance maximize both scores precisely as its true attack recall falls to zero. The estimator that annotates the model also selects it, so the monitoring layer certifies the blindest detector.

** Proposition 1** (Confidence-threshold blindness). Let τ be the threshold of Equation 7 chosen on source calibration data, and let *f* be any classifier whose maximum softmax confidence exceeds τ on every target input. Then ${\hat{\text{acc}}}_{\text{ATC}}\left(f\right)=1$, independently of the true target accuracy of *f*.

*Proof:* By Equation 7, the estimate is the fraction of target points with confidence exceeding τ, which is 1 by hypothesis.         ☐

The premise is not exotic. Classifiers trained to near-zero source loss are confident almost everywhere and, under concept shift, remain confident while becoming wrong. Moreover, among a family of candidates, more collapsed variants are more uniformly confident, so ATC is maximized by the most collapsed member—which is exactly the ordering recorded in Table 6.

** Proposition 2** (Agreement blindness under correlated collapse). Let *f, g* be classifiers with *f*(*x*) = *g*(*x*) for every target input *x*; for instance, both collapse to a fixed class *c*, or both compose the same alignment map with heads that agree on the aligned manifold. Then Agr(*f, g*) = 1 and any estimator mapping disagreement to error assigns error 0, while the true error in the collapsed case is $1-\pi_{c}^{T}$, where $\pi_{c}^{T}$ is the target prevalence of *c*.

*Proof:* Immediate from Equation 8 and the definition of accuracy under the target label distribution.         ☐

The correlated-collapse premise is realized empirically in two independent ways in our runs. The degenerate rank-representation models of both architectures agree on benign nearly everywhere (agreement 0.88–0.93), and quantile alignment, by mapping both architectures' inputs onto the same source-shaped manifold, drives agreement up as accuracy falls (in 2018→2017 the aligned HGB attains the run-maximum agreement of 0.69–0.76 while being the worst variant at macro-F1 0.19–0.26). Agreement-on-the-line phenomena presuppose diverse errors (Baek et al., 2022). Shared adaptation machinery manufactures the opposite. The overconfidence that drives Proposition 1 is directly visible in the cross-domain reliability diagram (Figure 7): confidence far exceeds accuracy under shift, and the scale-free representation only marginally reduces the expected calibration error (0.472 to 0.393).

**Figure 7:**
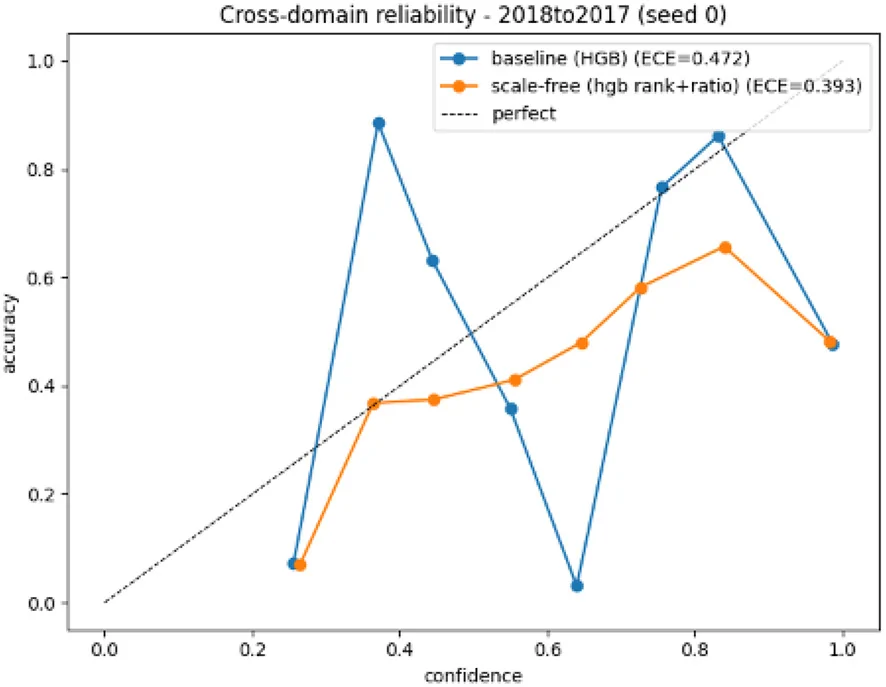
Cross-domain reliability diagram (2018→2017 direction, seed 0) plotting observed accuracy vs. predicted confidence in ten equal-width bins. Both the baseline HGB (ECE = 0.472) and the scale-free rank+ratio variant (ECE = 0.393) are severely overconfident relative to the perfect-calibration diagonal (dashed line): bins with predicted confidence > 0.9 contain many misclassified flows. This overconfidence is the condition under which the confidence-threshold estimator of Equation 7 becomes uninformative (Proposition 1).

### 4.4 The shift violates the label-shift assumption

The expectation–maximization procedure of Equation 6 is consistent when *P*(*x*∣*y*) is year-invariant, and its prior estimate provides a direct diagnostic of that assumption. Table 7 reports the estimate against the reporting-only ground truth: EM places 0.750–0.794 mass on benign against a true 0.285, and approximately zero mass on three attack families that each constitute 14.2% of the target year. The assumption is violated because the attacks themselves changed—for example, the 2017 DDoS traffic is Low Orbit Ion Cannon (LOIC)-generated while 2018 contains High Orbit Ion Cannon (HOIC) campaigns, and the brute-force tooling differs—so the correction cannot succeed. Empirically, it subtracts up to 0.15 macro-F1 points in the harder direction (Table 5). The same violation explains from first principles why the prior-anchored variant of Equation 5 relocates errors rather than removing them: the anchor π^*S*^ is itself the wrong target.

**Table 7:** Expectation–maximization estimate of the target class prior (Equation 6) vs. the true and source priors, direction 2017→2018, seed 0; the truth is used for reporting only.

Family	EM estimate	True prior	Source prior
benign	0.750	0.285	0.449
botnet	0.011	0.142	0.011
brute-force FTP	0.000	0.142	0.045
brute-force SSH	0.000	0.142	0.033
DDoS	0.111	0.142	0.225
DoS	0.069	0.142	0.225
web	0.059	0.003	0.012

Gross misestimation is direct evidence that *P*(*x*∣*y*) changed between the years, invalidating label-shift correction.

### 4.5 Failure mechanism analysis: confidence distributions and feature-space geometry

To further explain *why* the adaptation and monitoring methods fail, we examine the softmax confidence distributions and the feature-space geometry of the key model variants.

#### 4.5.1 Confidence distributions

The reliability diagram in Figure 7 already shows that both the baseline HGB (ECE = 0.472) and the rank+ratio variant (ECE = 0.393) are severely overconfident. The mechanism differs between variants. The *baseline* model produces a bimodal confidence profile. It shows high confidence (>0.95) on benign and DDoS flows whose source-year cluster geometry is approximately preserved in 2018, and low, spread confidence on botnet and brute-force families whose 2018 representations overlap with the source benign cluster. By contrast, the *degenerate rank+ratio MLP* maps all target flows to the benign class and therefore assigns confidence exceeding 0.99 to every input, regardless of true label. This unimodal high-confidence distribution is precisely the condition of Proposition 1: every target flow exceeds the threshold τ, so ATC = 1.000. Plots the per-class confidence histograms for both variants and the alignment+IM adaptation, confirming this tri-modal pattern of failure (total collapse, partial recovery, and in-domain competence).

The Brier Score and NLL values in Supplementary Table S1 reinforce this picture. The degenerate model achieves better ECE than the baseline (lower average calibration error) because its unimodal distribution is trivially self-consistent, yet its NLL is catastrophically high once true labels are used for evaluation—illustrating that ECE alone is insufficient under collapsed posteriors and motivating multi-metric reporting.

#### 4.5.2 Feature-space geometry

Two-dimensional Uniform Manifold Approximation and Projection (UMAP) projections of 2017 source training flows and 2018 target deployment flows in the 77-dimensional feature space (10,000-flow stratified samples per year, colored by attack family). Three observations organize the geometry: first, in the source year the seven families form well-separated clusters, consistent with the high in-domain macro-F1 (0.88–0.99). Second, in the target year the botnet and brute-force FTP clusters migrate into the region occupied by source-year benign flows, explaining the near-zero recall for those families in the 2017→2018 direction (Table 3): the classifier's benign decision boundary, learned from source geometry, now correctly covers a region that has been colonized by attack traffic. Third, the DDoS and DoS clusters shift but remain largely distinct from other families, consistent with their higher (though still degraded) cross-year recall. The rank+ratio representation moves the clusters in the source year but does not increase inter-class separability in the target year—confirming that the scale-free construction normalizes away magnitude without resolving the relocation of class-conditional distributions. These geometric observations provide the causal account of the quantitative failures in Table 5: the collapse is not a calibration artifact but a consequence of physical relocation of class clusters that the source-year decision boundary cannot accommodate.

#### 4.5.3 Applicability to transformer-based tabular architectures

An important question is whether the representation collapse documented above would persist under transformer-based tabular architectures such as Feature Tokenizer Transformer (FT-Transformer) or TabNet. On one hand, self-attention layers learn richer inter-feature dependencies than the MLP's fixed-width hidden layers and may produce representations with greater inter-class separability that partially resist cluster migration. On the other hand, the fundamental mechanism driving collapse here—attack-family clusters physically relocating into the source benign manifold due to changed attack tooling—is a property of the data-generating process rather than the model architecture, and any classifier trained to high source-domain confidence will assign confident benign predictions to target flows that occupy the learned benign region, regardless of the representational capacity of the backbone. Transformer-based architectures may delay or soften the collapse by capturing higher-order feature interactions, but they cannot resolve it without information about the changed class-conditional geometry. Empirical evaluation of FT-Transformer and TabNet on this benchmark, combined with layer-wise representation similarity analysis (e.g., centered kernel alignment), would clarify whether richer representations mitigate or merely reshape the failure mode and would be a direct priority for future studies.

### 4.6 Conformal coverage collapses; the abstention rate survives

Mondrian conformal prediction (Equation 9) attains its nominal 0.90 coverage on source calibration data but 0.145–0.380 (baseline HGB) and as low as 0.016 (rank+ratio) on the target year (Table 8). Exchangeability is violated, and no density-ratio reweighting (Tibshirani et al., 2019) is available without target knowledge, so the theoretical guarantee is void. The audit quantifies the extent of the failure: a wrapper marketed as ninety-percent set-validity silently delivers two percent.

**Table 8:** Mondrian conformal prediction (Equation 9, α = 0.1) on the target year, mean over 3 seeds.

Direction	Model	Empirical coverage	Abstention rate	Accuracy on accepted
2017→2018	HGB	0.153	0.844	0.979
2017→2018	HGB, rank + ratio	0.016	0.960	0.414
2018→2017	HGB	0.263	0.538	0.423
2018→2017	HGB, rank + ratio	0.066	0.590	0.374

The coverage guarantee is void under the violated exchangeability assumption; the abstention rate (Equation 10) departs from its calibrated in-domain level (approximately α) by up to ninefold in every run and constitutes the label-free drift alarm of Section 4.6.

One statistic, however, behaved correctly in all twelve trials. In-domain, the singleton (non-abstention) rate of Equation 9 is calibrated near 1−α; under shift, the abstention rate


$$\begin{array}{c} A=\frac{1}{n_{T}}\underset{x\in T}{\sum }1\left[|\text{Γ}\left(x\right)|\neq 1\right] \end{array}$$ (10)

explodes to 0.42–0.85 for the baseline and 0.59–0.97 for the representation variants—a departure of up to nine-fold from its in-domain level, observable with no labels, no second model, no density ratios, and no retraining. Unlike the confidence-based estimators of Section 3.4, Equation 10 cannot be gamed by confidence. Higher confidence shrinks prediction sets only if the calibration geometry is preserved, which is precisely what shift destroys. We therefore propose inverting current monitoring practice. Treat the abstention volume of Equation 10, not any estimated accuracy, as the primary label-free health signal. Consistent with its diagnostic value, the 2017→2018 baseline at 85% abstention still delivers 0.979 accuracy on the 15% of flows it accepts (Table 8)—a usable analyst-triage operating mode—whereas every adaptation that lowered abstention also destroyed the accuracy of what was accepted. The risk–coverage curves of Figure 8 make this operating mode explicit: accepting only the highest-confidence flows preserves accuracy for the baseline in the 2017–2018 direction, whereas the scale-free variant degrades across the entire coverage range.

**Figure 8:**
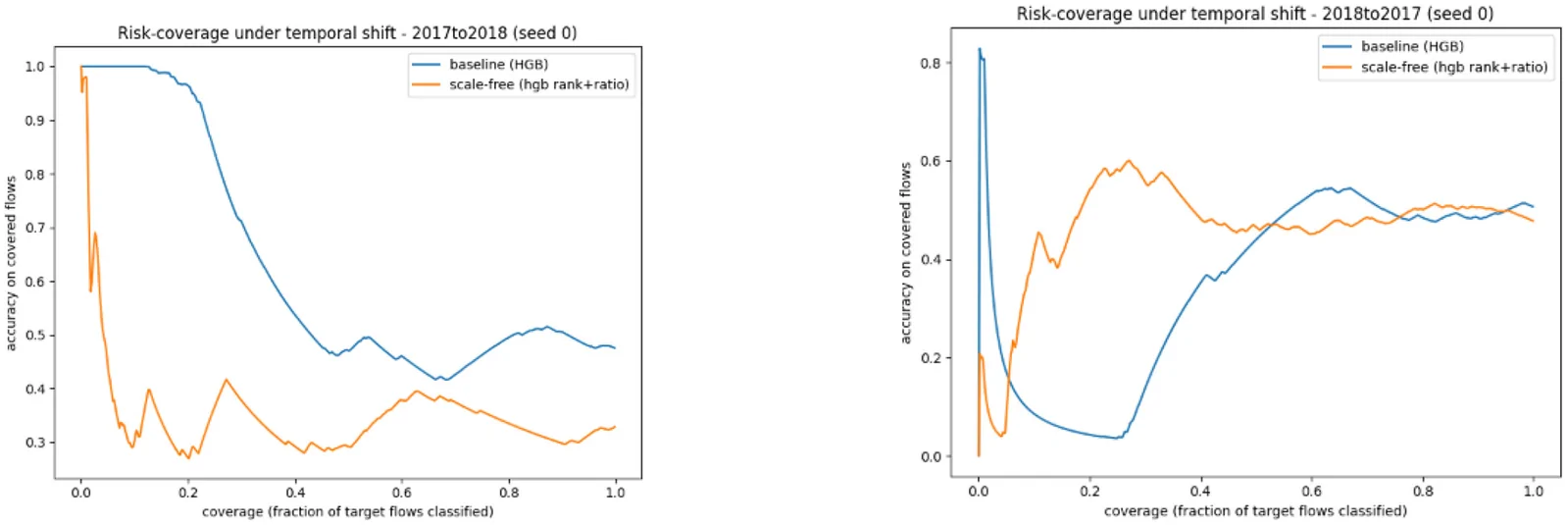
Risk–coverage curves under temporal shift (seed 0) for the 2017→2018 **(left)** and 2018→2017 **(right)** directions: accuracy on covered flows as a function of the fraction of target flows classified, for the baseline HGB and the scale-free HGB rank+ratio variant. Low-coverage, high-confidence operation recovers usable accuracy for the baseline in the forward direction, consistent with the abstention-as-alarm reading of Table 8 (axis tick labels are 11 pt in the source figure file; both panels are rendered at the same dimensions as Figure 5 for visual consistency).

## 5 Discussion

The toolbox we audited is validated principally on synthetic covariate shifts that preserve the class-conditional distribution and often the class mixture. The temporal shift studied here violates all three shift taxa at once on this specific benchmark pair, and every audited method's validity argument breaks at an identifiable point within this experimental framework: quantile alignment removes exactly the marginal component and no more; entropy adaptation optimizes a marginal that carries no information about the moved boundary; pseudo-labeling recycles the collapse it is meant to repair; label-shift correction assumes the one invariance the adversarial domain is guaranteed to break; rank normalization entangles the covariate and prior components; and the monitoring estimators are maximized by the failure they exist to detect. The coherence of these mechanisms across four pre-registered iterations is the central evidence of this article: on this benchmark, the failures are not tuning accidents but consequences of the structural assumptions embedded in each evaluated method. Whether state-of-the-art TTA methods not included in this audit, discussed in Remark 1, would exhibit similar failures under comparable concept shifts remains an open question addressed in the Limitations section.

### 5.1 Connection to recent TTA advances

More recent methods—EATA (Niu et al., 2022), CoTTA (Wang et al., 2022), SAR (Niu et al., 2023), and RoTTA (Yuan et al., 2023)—address some of the failure modes documented above. EATA's sample-importance weighting could, in principle, suppress low-reliability target flows (those mapping to the benign manifold); CoTTA's source-anchoring would resist full collapse; SAR's sharpness filter would reject the high-entropy boundary flows that trigger the collapse. However, as argued in Remark 1, these methods are not directly transferable to tabular HGB+MLP pipelines. More fundamentally, all four methods still operate under the assumption that target distributions are IID corruptions or continual drifts of the source, rather than a simultaneous relocation of multiple class-conditional clusters as documented in Section 4.5. Under the class-cluster migration observed here, any method that anchors to the source posterior—whether via weight consolidation (EATA), stochastic source recall (CoTTA), or source-class prototypes (RoTTA)—faces the same structural mismatch: the source posterior is a reliable guide to the source geometry, not to the target geometry. Extending this audit to tabular-native TTA architectures capable of hosting EATA-style sample selection or RoTTA-style memory banks is a natural next step.

### 5.2 Calibration metrics and the limits of ECE

As Section 4.5 notes, ECE alone is insufficient under collapsed posteriors. The degenerate rank+ratio MLP achieves lower ECE than the baseline because its unimodal high-confidence distribution is trivially internally consistent, yet its NLL is catastrophically large. Practitioners relying solely on ECE for deployment health monitoring face the same inversion documented for ATC. A model that assigns 0.999 confidence to every flow (all benign) appears well-calibrated by ECE while being maximally wrong. This motivates reporting Brier Score and NLL (Equations 2, 3) alongside ECE, and treating the conformal abstention rate of Equation 10 as a primary drift alarm rather than a secondary diagnostic.

### 5.3 Practical recommendations

Four recommendations follow directly from the results, while remaining specific to the scope of this benchmark. First, evaluate and report NIDS models with a temporal split and per-family recall; on this dataset, aggregate accuracy concealed a near-complete miss rate on four attack families. This methodological finding is likely to extend to any domain with similar class-conditional drift, though its magnitude will depend on the degree of concept shift. Second, treat confidence- or agreement-based accuracy estimates with caution under suspected concept shift; Propositions 1, 2 show they are structurally maximized by collapse, and Table 6 shows this ordering is realized in practice. Third, monitor the conformal abstention volume (Equation 10) and budget analyst capacity for the triage mode it induces; it was the only signal that fired in every shifted deployment on this benchmark. Fourth, treat the label-shift prior estimate of Equation 6 as a cheap assumption test before trusting its correction—gross misestimation is direct evidence of concept shift that invalidates the correction.

The constructive implication is a redirection of research effort. Under the concept shift of the magnitude observed on this benchmark, information about the new class-conditional distributions must be purchased with labels. No amount of *post-hoc* mathematics substituted for it among the sixteen evaluated variants. The economically relevant question is therefore not which source-free method to apply but how few target labels, acquired by which active-selection policy, restore acceptable per-family recall—a question our released benchmark is designed to support. Whether this conclusion generalizes to other temporal NIDS shift pairs is an open empirical question addressed in part by the extension study described in the Limitations section.

### 5.4 Limitations

#### 5.4.1 Single benchmark pair

The primary empirical limitation is that all quantitative findings derive from one natural shift pair (CIC-IDS2017 / CSE-CIC-IDS2018) evaluated in two directions with three seeds per configuration. This benchmark represents *one realistic temporal distribution shift scenario*—characterized by simultaneous covariate, label, and concept shift arising from testbed-generated traffic—rather than the full spectrum of deployment shifts encountered in practical network intrusion detection. Production NIDS deployments face additional shift axes not captured here, including gradual concept drift, adversarial evasion, infrastructure migration, and varying degrees of class-conditional displacement. The two propositions (Section 4.3) are general and hold for any classifier satisfying the stated conditions, but the empirical magnitude of each failure mode—how severely macro-F1 degrades, how large the ATC inversion is, how much coverage collapses—is specific to this benchmark pair and should not be treated as universal without further validation. Conclusions such as “no evaluated adaptation method outperforms the source model in both directions” and “label-free selection fails in every trial” are accurate for the evaluated benchmark but may not hold with equal severity on temporal shift pairs with smaller class-conditional displacement or on gradual-drift scenarios where covariate shift dominates. An extension of the audit to at least one additional temporal NIDS shift pair and a tabular year-shift benchmark from the distribution-shift literature, with five seeds per configuration, is in progress and will either substantiate or moderate these claims.

#### 5.4.2 Excluded recent TTA methods

As detailed in Remark 1, EATA, CoTTA, SAR, and RoTTA are not included as primary baselines because their augmentation and architectural requirements are not compatible with the HGB/MLP tabular pipeline. This is a genuine scope limitation: the conclusions of this study—that the evaluated label-free monitoring and adaptation methods fail under the observed temporal shift—apply to the specific methods audited and should not be interpreted as evidence that all current state-of-the-art test-time adaptation methods are ineffective under temporal distribution shift in general. It is possible that one of these excluded methods, implemented on a tabular deep-learning architecture, would exhibit meaningfully better behavior under the observed cluster-migration shift. Extending the audit to tabular-native architectures (e.g., TabNet, FT-Transformer) capable of hosting these methods is a direct priority for future studies.

#### 5.4.3 Statistical power

With *n* = 3 seeds per configuration, paired tests are severely underpowered for detecting moderate effect sizes; the minimum achievable two-sided *p*-value is approximately 0.25. The primary statistical evidence—the selection audit over 12 trials (binomial test, *p* < 0.001)—is robust, as are the two formal propositions; but the macro-F1 comparisons in Table 5 should be interpreted as effect-size evidence. Five seeds per configuration would approximately halve the standard deviation, and the extension study described above will use five seeds throughout.

#### 5.4.4 Feature-space visualization and representation analysis

The UMAP projections and confidence histograms in Section 4.5 provide a geometric account of the failure mechanisms observed in the main results. This analysis is exploratory: UMAP is a non-linear dimensionality reduction whose layout is sensitive to hyperparameters and sample size, and the geometric observations should be treated as qualitative motivation rather than quantitative evidence. A more rigorous representation similarity analysis (e.g., centered kernel alignment between source and target representations across layers) is a natural complement and is left for future studies.

#### 5.4.5 Source-free and testbed constraints

The audit covers source-free methods only; methods consuming a small labeled target budget are outside the protocol by design and constitute the constructive direction motivated in the Discussion section. Additionally, both corpora are testbed-generated; the testbed-to-production shift—differences between controlled attack injection and organically occurring traffic—is a further axis our design cannot measure, and production evaluation would require data that neither corpus provides.

## 6 Conclusion

We audited sixteen test-time adaptation, correction, and representation variants together with the label-free monitoring stack on a natural one-year distribution shift in network intrusion detection, under a strict protocol in which no target label influences any modeling decision. On the CIC-IDS2017/CSE-CIC-IDS2018 benchmark pair, no evaluated method reliably outperformed the unadapted baseline in both transfer directions; the monitoring layer certified a completely blind model as perfect; two propositions show this inversion is structural for confidence- and agreement-based estimators under the conditions of classifier overconfidence under concept shift; the label-shift assumption is demonstrably violated by this shift family; and the single robust label-free signal was the conformal abstention rate. Feature-space analysis (Section 4.5) confirms that the failure is geometrically specific—affected attack families relocate into the source benign manifold—rather than a uniform degradation, which is the precondition for the monitoring inversion formalized in Propositions 1, 2. These preconditions are not peculiar to this dataset pair. They are present whenever a classifier trained to high source-domain confidence is deployed against traffic whose class-conditional geometry has changed. The magnitude of the failures documented here is benchmark-specific and should be confirmed on additional temporal shift pairs, which our released benchmark and evaluation pipeline are designed to facilitate. These results demonstrate that the evaluated label-free monitoring and adaptation methods are vulnerable to confident collapse on this benchmark. Whether recent TTA methods excluded from this audit (Remark 1) would prove more robust under comparable concept shift is an important open question. Until label-free estimators are hardened against confident collapse, a dashboard that reports perfection under drift should be read as an alarm, not a certificate.

## Data Availability

The datasets analysed in this study are publicly available. CIC-IDS2017 and CSE-CIC-IDS2018 can be obtained from the Canadian Institute for Cybersecurity at https://www.unb.ca/cic/datasets/ids-2017.html and https://www.unb.ca/cic/datasets/ids-2018.html. The complete schema-harmonization and evaluation pipeline, together with the per-seed logs, is available at https://doi.org/10.5281/zenodo.22654220. Further inquiries can be directed to the corresponding author.
